# Nonreciprocal magnetoacoustic waves with out-of-plane phononic angular momenta

**DOI:** 10.1126/sciadv.ado2504

**Published:** 2024-07-10

**Authors:** Liyang Liao, Fa Chen, Jorge Puebla, Jun-ichiro Kishine, Kouta Kondou, Wei Luo, Degang Zhao, Yue Zhang, You Ba, Yoshichika Otani

**Affiliations:** ^1^ Institute for Solid State Physics, University of Tokyo, Kashiwa 277-8581, Japan.; ^2^ School of Integrated Circuits, Huazhong University of Science and Technology, Wuhan 430074, China.; ^3^ Center for Emergent Matter Science, RIKEN, Wako, Saitama 351-0198, Japan.; ^4^ The Open University of Japan, Chiba 261-0013, Japan.; ^5^ Quantum Research Center for Chirality, Institute for Molecular Science, Aichi 444-8585, Japan.; ^6^ School of Physics, Huazhong University of Science and Technology, Wuhan, Hubei 430074, China.; ^7^ Trans-scale Quantum Science Institute, University of Tokyo, Tokyo 113-8654, Japan.

## Abstract

Surface acoustic wave (SAW) can carry phononic angular momentum, showing great potential as an energy-efficient way to control magnetism. Still, out-of-plane phononic angular momentum in SAW and its interaction with magnetism remain elusive. Here, we studied the SAW-induced magnetoacoustic waves and spin pumping in Ni-based films on LiNbO_3_ with selected SAW propagation direction. The crystal inversion asymmetry induces circularly polarized phonons with large out-of-plane angular momenta so that up to 60% of the SAW power attenuates nonreciprocally controlled by the out-of-plane magnetization component. The SAW propagation direction dependence of the nonreciprocity verifies the crystal origin of the phononic angular momentum, and a chiral spin pumping demonstrates that the circular polarization can control the spin current generation efficiency. These results provide an additional degree of freedom for the acoustic control of magnetism and open an avenue for applying circularly polarized phonons.

## Teaser

Out-of-plane phononic angular momenta are observed in surface acoustic waves, enriching the acoustic control of magnetization.

## INTRODUCTION

Symmetry engineering plays a key role in spintronics for manipulating magnetization with high energy efficiency. In spin-orbit torque devices, by introducing spin sources with broken crystal inversion symmetry (*1*, *2*) or time-reversal symmetry (*3*, *4*), it becomes possible to achieve out-of-plane spin polarization. The out-of-plane spin polarization relying on symmetry engineering not only induces field-free switching of magnets with perpendicular anisotropy (*4*–*6*) but also reduces the power consumption of the magnetic memory devices (*7*, *8*).

Besides electron spins, other degrees of freedom capable of efficiently generating angular momenta also hold potential for manipulating magnetization. Recently, the coupling between surface acoustic wave (SAW) devices and magnetism has been gaining much attention (*9*), as SAW can be excited by electric fields in piezoelectric substrates without Joule heating. The coupling between SAW and magnetism induces acoustic ferromagnetic resonance (FMR) (*10*–*12*), nonreciprocal SAW transmission (*13*–*17*), acoustic spin-transfer torque (*18*, *19*), acoustic spin Hall magnetoresistance (*20*), and skyrmion creation (*21*) and manipulation (*22*). However, all these works are based on the in-plane angular momentum carried by Rayleigh-type SAW (*18*, *23*, *24*) or the compressional strains without angular momentum (*11*, *12*), limiting the flexibility of controlling out-of-plane magnetization through SAW. Out-of-plane phononic angular momentum in SAW and its coupling with magnetism remain elusive.

Similar to the spins in inversion symmetry-breaking spin Hall materials, phononic angular momentum can also be controlled by crystal symmetry (*25*–*29*). Thermally generated out-of-plane phononic angular momentum in inversion symmetry-breaking materials has been demonstrated to excite magnetization dynamics (*30*). Here, we explore the role of the crystal inversion symmetry breaking in SAW. We studied the SAW transmission and spin pumping in ferromagnetic stacks on a 128° Y-LiNbO_3_ with SAW propagation direction tilted from the *X* axis. A highly nonreciprocal magnetoacoustic wave controlled by the out-of-plane magnetization component show that the broken crystal symmetry can introduce circularly polarized acoustic phonons with out-of-plane angular momenta.

**Fig. 1.**
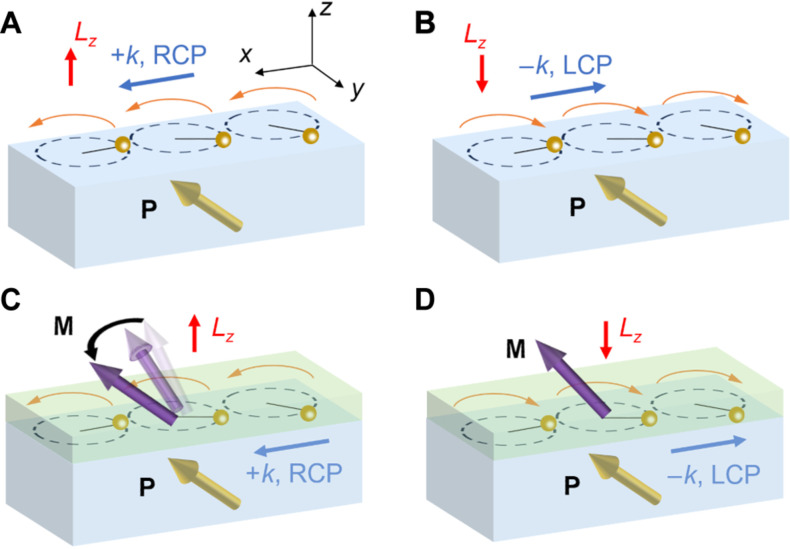
Schematic of circularly polarized phonons with out-of-plane angular momenta and asymmetric excitation of FMR. In a ferroelectric substrate, the *xz* mirror symmetry is broken and phonons can be circularly polarized. +*k* phonon has RCP and positive angular momentum *L_z_* (**A**), while −*k* phonon has LCP and negative angular momentum *L_z_* (**B**). When the substrate is capped by a magnetic thin film with an out-of-plane magnetization component, phonons with positive angular momentum *L_z_* can efficiently excite the FMR (**C**), while phonons with negative angular momentum *L_z_* can hardly excite the FMR (**D**).

As shown in Fig. 1 (A and B), with a spontaneous bulk ferroelectric polarization **P** breaking the *xz* mirror symmetry, ±*k* phonons are allowed to have right-hand circular polarization (RCP) and left-hand circular polarization (LCP) (*31*), carrying out of plane angular momentum ±*L_z_*. With a ferromagnetic Ni layer grown on the substrate, because FMR is right-handed, it can be excited efficiently by the RCP phonons (Fig. 1C) but not by the LCP phonons (Fig. 1D), with an upward magnetization component. We experimentally observed a highly nonreciprocal magnetoacoustic wave, with up to 60% of the SAW power attenuating nonreciprocally because of the asymmetric excitation of the FMR, revealing a strong circular polarization of the phonons. The nonreciprocal attenuation shows propagation direction dependence clearly following the crystal symmetry of the LiNbO_3_. When attaching Ni to a Pt layer, we observed a chiral spin pumping (*32*), where the phononic circular polarization controls the efficiency of the spin current generation. Our observation shows that because of the surface electric field distribution combining with the symmetry of the piezoelectric effect, circularly polarized phonons with out-of-plane angular momenta can be generated in gigahertz frequency, providing opportunities for the SAW-magnetism interactions and the application of circularly polarized phonons.

## RESULTS

### SAW-driven FMR with out-of-plane phononic angular momentum

Nonzero phononic angular momentum exists in acoustic systems with symmetry breaking, which supports acoustic velocity fields with local elliptically polarized profiles. This phononic angular momentum per mass is described as (*33*)

$$L=\frac{1}{2\omega }\text{Im}\left[v^{*}\times v\right]$$ (1)

where ω is the acoustic wave frequency and **v** is a complex acoustic velocity field, which relates to the displacement field **u** by **v** = −*i*ω**u**. For SAW propagating along the *x* axis in the *xy* plane, it is known that the Rayleigh wave carries an in-plane transverse angular momentum *L_y_* (*18*, *23*) due to the elliptically polarized profile in the *xz* plane. When the acoustic velocity field has an elliptically polarized profile in the *xy* plane, i.e., the substrate plane, an out-of-plane angular momentum *L_z_* will emerge (Fig. 1, A and B).

The coupling between magnetism and SAW is mainly caused by the magnetoelastic interaction between magnetization and strains (*10*–*12*, *15*). SAW thus applies an effective field on the magnet, which can have the same helicity with the SAW (carrying an angular momentum). As only right-handed magnetization precession is allowed, the helicity mismatch between the SAW and the magnetization precession leads to FMR excitation with different amplitudes (*13*–*16*). Equivalently, the magnetoelastic interaction transfers phononic angular momentum **L** into the spin system **S** (*18*), i.e., applying a torque (*34*) and generating magnons. The phonon-magnon scattering efficiency is different for **L**·**S** > 0 and **L**·**S** < 0, leading to a different magnetization precession amplitude. As a result, +*k* and −*k* Rayleigh SAWs with opposed circular polarization in the *xz* plane, or carrying ±*L_y_*, can be attenuated with different amplitudes when passing through a magnet, known as nonreciprocal SAW transmission (*13*–*16*).

For the coupling between magnetism and the *xy* plane–circularly polarized SAWs with *L_z_*, we first present our concept in a two-dimensional model (detailed in section S1), which captures the main physics in the magnetic thin film on LiNbO_3_, as will be shown via experiment and COMSOL simulation in the following. The two-dimensional model considers an in-plane displacement **u** = (*u_x_*, *u_y_*) and strains ε*_xx_* and ε*_xy_*. To harvest the coupling due to these two strains, it is necessary to rotate the magnetization **M** by angle θ in the *xz* plane from the *z* axis (*35*), allowing nonzero SAW-induced effective field components perpendicular to the magnetization. When 0° < θ < 90°, the circularly polarized phonon with *L_z_* leads to different FMR excitation efficiencies for the +*k* and ─*k* SAWs or an asymmetric excitation of FMR (see detailed derivation in section S1)

$$\Delta P_{\text{chiral}}\equiv \Delta P_{+k}-\Delta P_{-k}=\frac{2\alpha \gamma b^{2}}{M_{S}}\frac{\omega^{2}\omega_{F}k^{2}L_{z}\text{sin}2\theta \text{sin}\theta }{{\left({\omega_{F}}^{2}-\omega^{2}\right)}^{2}+\alpha^{2}{\omega_{F}}^{2}\omega^{2}}$$ (2)

where *k* is the wave vector of the SAW, Δ*P*_±*k*_ is the absorbed power due to the excitation of FMR induced by the ±*k* SAW, γ is the gyromagnetic ratio, α is the Gilbert damping, *M_S_* is the saturated magnetization, ω*_F_* is the FMR frequency, and *L_z_* is the out-of-plane phononic angular momentum given by Eq. 1. The different FMR amplitude is due to the helicity mismatch effect in the *xy* plane (section S1) or, equivalently, the angular momentum transfer efficiency difference when **L**·**S** > 0 and **L**·**S** < 0, from which a sin2θsinθ dependence the same as Eq. 2 can be derived (section S2).

The asymmetric excitation of FMR, manifested as a nonreciprocal SAW transmission, can be controlled by the circular polarization of the phonons, reflecting the out-of-plane angular momentum *L_z_*. The circular polarization in the *xy* plane leads to a distinct symmetry of the nonreciprocity from the conventional nonreciprocity for the Rayleigh waves: The reversal of the in-plane component by θ → −θ does not change the nonreciprocity, whereas the reversal of the out-of-plane component by θ → π −θ leads to an opposite nonreciprocity.

### Nonreciprocal magnetoacoustic waves

**Fig. 2.**
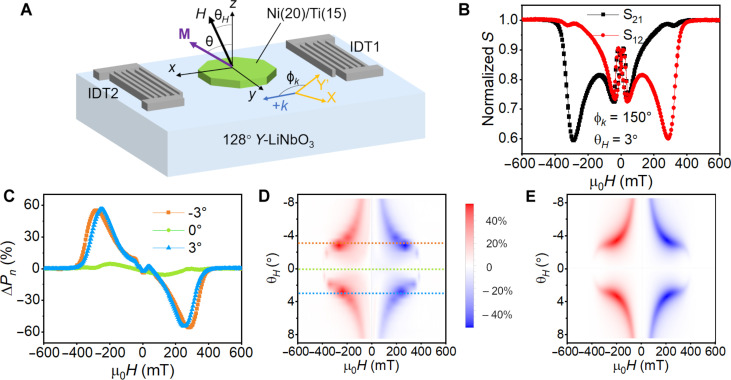
Nonreciprocal SAW transmission due to asymmetric excitation of FMR. (**A**) Schematic of the measurement setup. SAW with a wavelength of 2 μm and a frequency of 1.785 GHz is sent by IDTs to a Ni (20)/Ti (15) film (thickness in nanometers). The wave vector *k* is rotated with an angle ϕ*_k_* from the crystal *Y*′ axis of the 128° *Y*-cut LiNbO_3_. The film normal is defined as the *z* axis, and the wave vector direction is defined as the *x* axis. Note that the *x* and *y* axes in the device coordinate system are different from the *X* and *Y*′ axes in the crystal coordinate system. Magnetic field *H* is applied in the *xz* plane with an angle θ*_H_* from the *z* axis, resulting in the magnetization in the *xz* plane with an angle θ from the *z* axis. (**B**) The normalized transmission S_12_ and S_21_ as functions of the applied field at θ*_H_* = 3° in a device with ϕ*_k_* = 150°. (**C**) Nonreciprocal absorption Δ*P_n_* = (Δ*P*_+*k*_–Δ*P*_−*k*_)/*P* as a function of the applied field at θ*_H_* = 3°, 0°, and −3° in the device with ϕ*_k_* = 150°. (**D**) Color plot of Δ*P_n_* as a function of the applied field magnitude and angle θ*_H_*, where θ*_H_* = 3°, 0°, and −3° are marked by dash lines in the same colors as in (C). (**E**) Calculated Δ*P*_chiral_ (in arbitrary unit) as a function of the field magnitude and angle θ*_H_*.

Figure 2A shows the geometry of the SAW devices used in this experiment (detailed in section S2). SAW propagation direction is defined as the *x* axis, which is rotated by an angle of ϕ*_k_* from the crystal *Y*′ axis on the substrate plane. The SAW wavelength is 2 μm, with a frequency of 1.785 GHz. The substrate normal is defined as the *z* axis. Under a magnetic field tilted from the *z* axis in the *xz* plane by angle θ*_H_*, the magnetization **M** is drawn out of plane, with an angle θ > θ*_H_* from the *z* axis due to the easy-plane anisotropy. The transmission between the two interdigital transducers (IDTs) S_12_ and S_21_, corresponding to the +*k* and −*k* SAWs, respectively, are measured when scanning the magnetic field by a vector network analyzer with a time-gating technique. Figure 2B shows the S_12_ and S_21_ as a function of the magnetic field *H* along the out-of-plane angle θ*_H_* = 3° in a device with SAW propagating along the in-plane crystal angle ϕ*_k_* = 150°. The transmission parameters are normalized at 600 mT (~0.008 as shown in fig. S4), where the FMR frequency is much higher than the SAW frequency and no magnetism-related absorption happens. The main absorption peaks are located at ~±300 mT, determined by the FMR condition under the magnetic field and the magnetic anisotropy. A large difference between S_12_ and S_21_ is observed: At 300 mT, S_12_ is strongly suppressed, while S_21_ shows nearly no attenuation, corresponding to the different transmission between +*k* and −*k* SAWs, so-called nonreciprocity. While the +*k* SAW carrying positive *L_z_* can efficiently excite the FMR, the −*k* SAW carrying negative *L_z_* cannot, resulting in the strong and small attenuation of the S_12_ and S_21_, respectively. The situation reverses at −300 mT, where S_21_ is strongly attenuated and S_12_ still has a large value. The negative field leads to a downward magnetization component in the out-of-plane direction so that the −*k* SAW carrying negative *L_z_* can efficiently excite the FMR and be attenuated pronouncedly.

To compare the nonreciprocal transmission at different magnetic field angles, nonreciprocal absorption ratios Δ*P_n_* = (Δ*P*_+*k*_–Δ*P*_−*k*_)/*P* in the same device as a function of the magnetic field at different magnetic field angles θ*_H_* are plotted in Fig. 2C, where *P* is the total SAW transmission power (see Materials and Methods). For θ*_H_* = 3° and −3°, nonreciprocal absorption up to 60% is observed with the same sign, i.e., a negative (positive) Δ*P_n_* peak at the positive (negative) field. The symmetry is consistent with the nonreciprocity caused by the circularly polarized phonons with out-of-plane angular momentum as discussed in the previous section. When θ*_H_* = 0°, the magnetization **M** is fully out-of-plane at the FMR condition so that the in-plane strains ε*_xx_* and ε*_xy_* have little coupling with **M**. Thus, no obvious absorption exists at θ*_H_* = 0°.

Figure 2D further shows a color plot of Δ*P_n_* as a function of magnetic field magnitude *H* and angle θ*_H_* from −9° to 9°. It can be seen that for all angles, while the opposite field leads to opposite Δ*P_n_*, the reversal of the in-plane component by θ → −θ does not change Δ*P_n_*, i.e., the nonreciprocity is controlled by the out-of-plane magnetization component. For larger |θ*_H_*|, the blue and red patterns get closer to the center line, representing smaller resonance fields. This can be understood as that when the magnetic field and the magnetization are rotated toward the in-plane direction, a high magnetic field is no longer needed to overcome easy-plane anisotropy to reach the resonance condition. A calculated color plot of Δ*P*_chiral_ using Eq. 2 in the arbitrary unit is shown in Fig. 2E (detailed in section S4). It can be seen that the theoretical Δ*P*_chiral_ associated with the angular momentum *L_z_* well reproduces the features of the experimental Δ*P_n_*. The observed nonreciprocity hence evidences the angular momentum *L_z_* carried by the SAW.

### Crystal origin of the phononic angular momentum

**Fig. 3.**
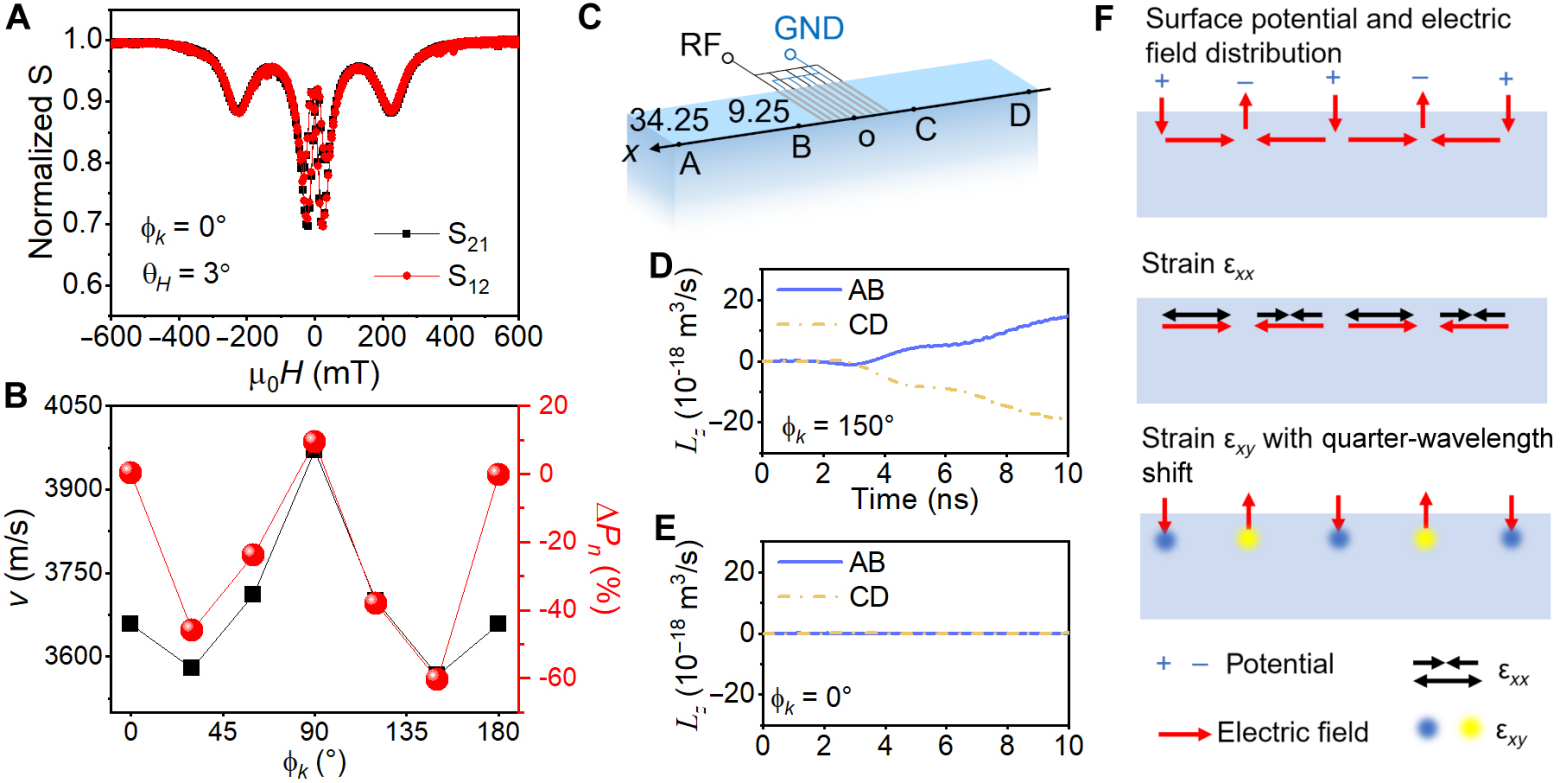
Propagation dependence of the SAW transmission and the crystal origin of the phononic angular momentum. (**A**) The normalized transmission S_12_ and S_21_ as functions of the applied field at θ*_H_* = 3° in a device with ϕ*_k_* = 0°. (**B**) SAW velocity *v*_SAW_ and peak nonreciprocal absorption as functions of ϕ*_k_*. (**C**) Setup of the COMSOL simulation. The coordinates of *A*, *B*, *C*, and *D* are 34.25, 9.25, −9.25, and −34.25 μm, respectively. By changing the propagation direction, the simulation gives the integrated *L_z_* in the area *AB* and *CD* as functions of time at ϕ*_k_* = 150° (**D**) and ϕ*_k_* = 0° (**E**). (**F**) Schematic of the piezoelectric coupling arousing the phononic angular momentum *L_z_*. The blue plus (+) and minus (−) signs label the electric potential. The red single-sided arrows label the electric field. The black double-sided arrows label the longitudinal strain ε*_xx_*, and the cloudy blue/yellow dots label the shear strain ε*_xy_*.

Having established the nonreciprocal SAW transmission in the device with SAWs propagating on the in-plane crystal angle ϕ*_k_* = 150°, we next clarify the origin of the out-of-plane angular momentum *L_z_*. To allow the *L_z_*, it is necessary to break the *xz* mirror symmetry (*1*). Because our devices have no artificial designs breaking this mirror symmetry, the symmetry breaking can only come from the piezoelectric substrate, which is naturally inversion-symmetry-broken because of the ferroelectricity. We hence study the propagation direction dependence of the nonreciprocity by fabricating six devices with SAWs propagating on the crystal in-plane angles ϕ*_k_* from 0° to 150° (step 30°), the same wavelength of 2 μm, and a frequency of 1.785 to 1.977 GHz, depending on the SAW velocities at different directions. Figure 3A shows the SAW transmission in the ϕ*_k_* = 0° device, with the same measurement setup in Fig. 2A at an out-of-plane magnetic field angle θ*_H_* = 3°. The S_12_ and S_21_ are almost identical, showing that no nonreciprocity exists in the device and no out-of-plane angular momentum *L_z_* is carried by the SAW along ϕ*_k_* = 0°. Further summarizing the SAW velocity *v*_SAW_ measured from the frequency of each device and the peak nonreciprocal absorption Δ*P_n_* as functions of ϕ*_k_* (Fig. 3B), it is found that *v*_SAW_ and Δ*P_n_* have similar ϕ*_k_* dependence. At ϕ*_k_* = 0° and 90°, *v*_SAW_ has the local maxima, and the *v*_SAW_ curve is approximately symmetric about the ϕ*_k_* = 90° axis. Meanwhile, the absolute value of Δ*P_n_* is zero at ϕ*_k_* = 0° and small at ϕ*_k_* = 90°, while it is large at other four intermediate angles. Because the ϕ*_k_* dependence of *v*_SAW_ is due to the crystal symmetry (*36*), the propagation direction dependence of the nonreciprocity and its close relationship with the evolution of *v*_SAW_ show that the nonreciprocity is also controlled by the crystal symmetry, and the angular momentum *L_z_* originates from the piezoelectric substrate with the *xz* mirror symmetry breaking.

To clarify the mechanism forming the phononic angular momentum, we performed a COMSOL simulation of the SAW excitation. As shown in Fig. 3C, we calculate the out-of-plane angular momentum *L_z_* at area *AB* and *CD*, accounting for the +*k* and −*k* SAWs, respectively (detailed in section S5). Figure 3D displays the integrated *L_z_* as functions of time at ϕ*_k_* = 150°. From *t* = 3 ns, as the SAW reaches the *B* and *C* points, nonzero *L_z_* emerges. *L_z_* increases as the SAW amplitude increases and propagates. The integrated *L_z_* values at *AB* and *CD* areas have a similar magnitude and opposite sign. When the propagation direction is rotated to ϕ*_k_* = 0°, *L_z_* is zero at all times, showing that no out-of-plane spin is carried by the SAW in this crystal direction, consistent with the absence of the nonreciprocity at ϕ*_k_* = 0°.

The emergence of the phononic angular momentum can be understood as depicted in Fig. 3F. Any SAW on a piezoelectric substrate that can be electrically generated accompanies a surface potential. The positive and negative potentials are staggered, with a period of the wavelength. The out-of-plane electric field *E_z_* is concentrated below the surface area with maximum positive and negative potentials, whereas the in-plane electric field *E_x_* always points from the positive potential to the negative one. Thus, the maximum position of *E_z_* and *E_x_* has a shift of a quarter wavelength. Meanwhile, different strain components may respond differently to *E_z_* and *E_x_*. Suppose the longitudinal strain ε*_xx_* is mainly excited by *E_x_* and the shear strain ε*_xy_* is mainly excited by *E_z_*, the two strains will then obtain a quarter-wavelength shift from the electric field distribution (section S6). Given that the displacement *u_x_* and *u_y_* depend on strain ε*_xx_* and ε*_xy_*, respectively, they will also get the same quarter-wavelength shift. In propagating waves, the quarter-wavelength shift is equivalent to a 90° phase shift, which causes a circular polarization and the out-of-plane angular momentum *L_z_*. The same quarter-wavelength shift leads to an opposite phase shift for opposite propagation direction, and therefore, SAWs with an opposite sign of *k* carry an opposite sign of *L_z_*.

Note that in real devices, a small ε*_zz_* also exists, with an opposite phase to ε*_xx_* according to the COMSOL simulation (section S5). To take the ε*_zz_* into account in the two-dimensional model presented above, one just needs to replace ε*_xx_* with a renormalized ε*_xx_′* = ε*_xx_* − ε*_zz_* (see section S5), which cannot change the symmetry of the nonreciprocity described by Eq. 2. As ε*_zz_* is generally much smaller than ε*_xx_*, the renormalized ε*_xx_′* would not substantially depart from ε*_xx_*, and all above analyses based on the hybridization between ε*_xx_* and ε*_xy_* should still be valid.

### Chiral spin pumping driven by SAW

**Fig. 4.**
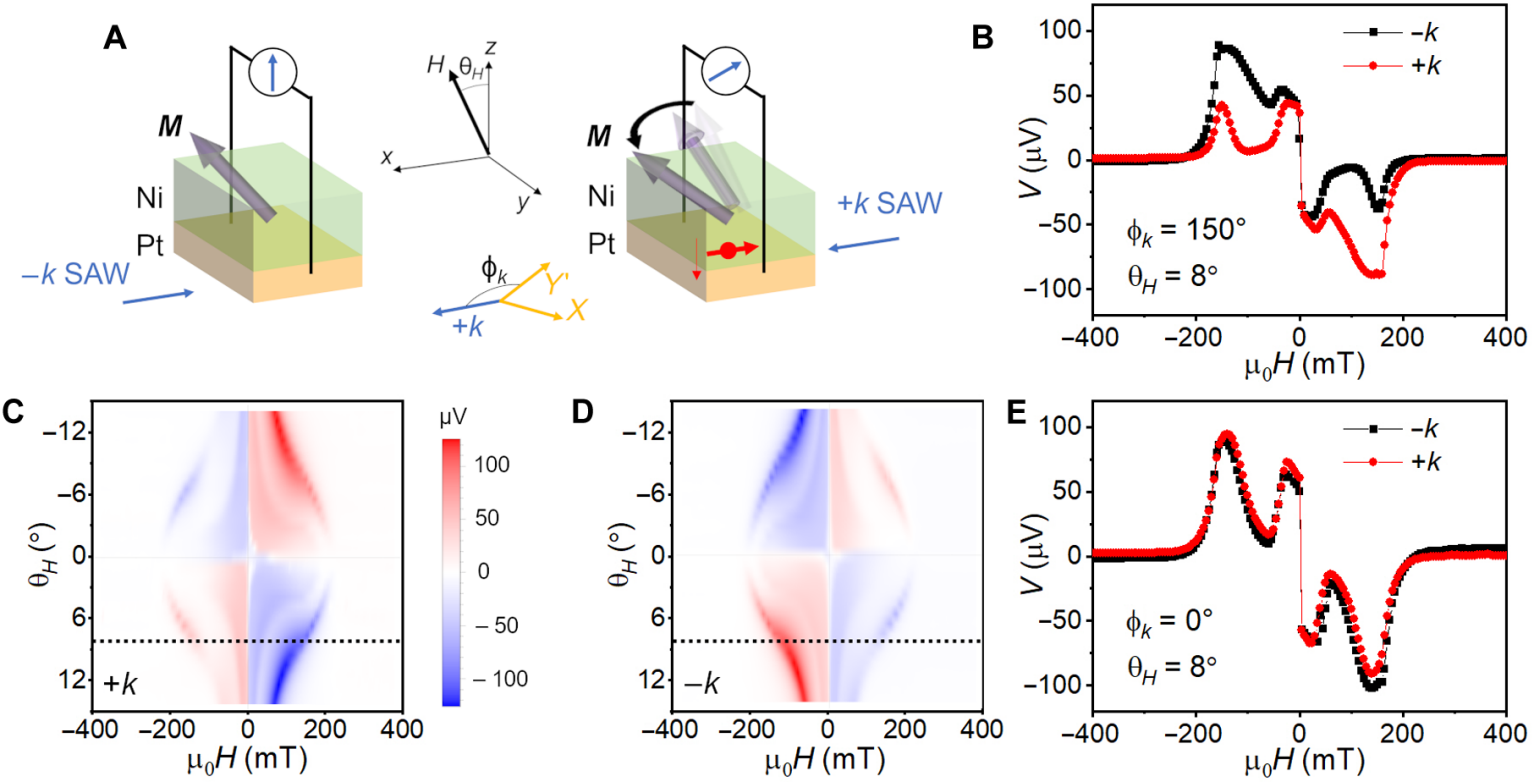
SAW-driven chiral spin pumping in a Pt(4)/Ni(14) heterostructure. (**A**) Schematic of the chiral spin pumping. Sending ±*k* SAWs along ϕ*_k_* = 150° to a Pt (4)/Ni (14) heterostructure capped by 2-nm Ti, FMR is excited efficiently by the +*k* SAW but not by the −*k* SAW. FMR drives spin pumping into the Pt layer, and a DC voltage can be generated via the ISHE. Hence, a larger DC voltage is obtained with the +*k* SAW. (**B**) DC voltage *V* across the Pt (4)/Ni (14) strip as a function of the applied field with ±*k* SAW. The field is along θ*_H_* = 8°, and the device has ϕ*_k_* = 150°. Color plots summarize *V* as a function of the applied field magnitude and angle θ*_H_* with +*k* SAW (**C**) and −*k* SAW (**D**), where θ*_H_* = 8° used in (B) is marked by black dash lines. (**E**) DC voltage *V* as a function of the applied field with ±*k* SAW in a device with ϕ*_k_* = 0°.

We lastly applied the asymmetric excitation of FMR in generating spin current, which can be detected via inverse spin Hall effect (ISHE) in a Pt layer adjacent to the Ni layer. The magnetic field is kept in the *xz* plane with an out-of-plane angle θ*_H_* from the *z* axis. Hence, the projection of the in-plane magnetization is along the *x* axis, and the ISHE geometry (*10*) requires an ISHE voltage measured along the *y* axis. Because the FMR efficiency is controlled by the phonon circular polarization, it can be expected that the spin pumping efficiency follows the same principle: Spin current generation by the RCP phonons is more efficient than that by the LCP phonons (Fig. 4A). The ISHE voltage, proportional to the spin current, is therefore large when sending the +*k* SAW with the RCP phonons and small when sending the −*k* SAW with the LCP phonons.

Figure 4B plots the measured voltage *V* as a function of the applied field under ±*k* SAWs along the in-plane crystal angle ϕ*_k_* = 150°. The field direction is θ*_H_* = 8° to provide a larger in-plane magnetization component and a larger spin pumping signal. The signals under +*k* SAW and −*k* SAW show a remarkable difference: At ~160 mT, the negative resonance peak under +*k* SAW has a much larger absolute value than the one under −*k* SAW; at ~−160 mT, a positive resonance peak appears, and the situation is reversed, with the −*k* SAW exciting a much larger signal. The typical spin pumping voltage ~80 μV gives a magnetization precession angle of ~5° (*37*), still in the linear regime (*38*). The evolution of the spin pumping signal under different magnetic field magnitudes and directions are summarized in color plots Fig. 4, C and D, corresponding to the +*k* and −*k* SAWs, respectively. Under +*k* SAW (Fig. 4C), the voltage always has a larger absolute value at the positive field than at the negative field, showing a larger spin pumping efficiency when the RCP phonons drive the right-hand FMR. The sign change of the voltage associated with the sign change of θ*_H_* and the field is due to the ISHE geometry (*10*), so the same features appear under the −*k* SAW (Fig. 4D). However, the absolute value is larger at the negative field, as the LCP phonons can drive the right-hand FMR when the magnetization has a downward component. When the SAW propagation angle ϕ*_k_* is rotated to 0°, the spin pumping voltage has no distinguishable difference under ±*k* SAWs (Fig. 4E), consistent with the unpolarized phonons in this direction.

## DISCUSSION

We observed SAWs with circular polarization in the surface plane, carrying an out-of-plane angular momentum, evidenced by a nonreciprocity with a unique symmetry when coupling to a magnet: The nonreciprocity does not change sign when the in-plane magnetization component is reversed but changes sign when the out-of-plane component is reversed, in contrast to the conventional Rayleigh waves. The nonreciprocity can be understood from the helicity mismatch effect between the magnetoelastic effective field and the magnetization dynamic or, equivalently, the different phonon-magnon scatter rates via the magnetoelastic interaction due to the different angles between their angular momenta. Because such circular polarization in the surface plane requires only in-plane strains (ε*_xx_* and ε*_xy_*), the nonreciprocity survives in the thin-film limit. As a result, while the magnetoelastic nonreciprocity in Rayleigh wave is limited by the film thickness (*14*, *15*), large nonreciprocity (Fig. 2B) can be achieved in a thin Ni layer in our geometry, without using any sophisticated multilayer designs (*17*).

It is necessary to point out that the magnetoelastic interaction is not the only source to transfer angular momentum into the magnet, as the Zeeman energy from the external field and the easy-plane anisotropy of the magnet also contributes. While the angular momentum view discussed above applies to the SAW nonreciprocity and explains the sin2θsinθ dependence (section S2), one might not understand the SAW-magnetization interaction merely from the phononic angular momentum transfer; rather, it is a joint effect of the Zeeman energy, anisotropy and the magnetoelastic interaction. We also emphasize that the consistent angular momentum view and the helicity mismatch view are not unique for our out-of-plane geometry. As shown in (*18*), the angular momentum view also applies to the Rayleigh waves, which carry an in-plane angular momentum.

The SAWs that we used with an out-of-plane angular momentum can be regarded as a hybridized wave containing Rayleigh wave, a shear-horizontal wave, and some leakage into the substrate (section S5). While the basic properties of these SAWs have been reported in previous studies (*36*, *39*), the hybridized nature and the leakage make them less favorable for the SAW filter industry, and their potential of the circular polarization with the out-of-plane angular momentum is largely overlooked.

In conclusion, we have shown that the crystal inversion symmetry breaking can be used to generate circularly polarized phonons with out-of-plane angular momenta in SAW devices. The circularly polarized phonons couples to out-of-plane magnetization selectively, resulting in distinct FMR efficiencies determined by the phonon circular polarization and the out-of-plane magnetization component. Because of this asymmetric excitation of FMR, up to 60% of the incident SAW power is absorbed nonreciprocally. The phonon circular polarization arises from a joint effect of the piezoelectric interaction and the surface electric field distribution, and the crystal symmetry determines the phonon circular polarization through the piezoelectric matrix. We hence can control the circular polarization direction of acoustic phonons without using complex structures (*33*, *40*). As a result of the asymmetric excitation of FMR, a chiral spin pumping effect is observed, where the spin current generation efficiency is decided by the phonon circular polarization. The coherent coupling between the gigahertz magnetic dynamics and the circularly polarized phonons originating from the crystal symmetry opens opportunities for the circularly polarized phonons in spintronic applications. In particular, our results may advance the manipulation of magnetic objects with out-of-plane magnetization components, such as nanomagnets with perpendicular anisotropy (*4*, *41*), magnetic vortex (*42*), and skyrmions (*22*).

## MATERIALS AND METHODS

### Sample preparation

The SAW devices are fabricated on a commercial 128° *Y*-cut LiNbO_3_ substrate. The IDTs are defined by electron-beam (e-beam) lithography with a width of 400 nm and a separation of 600 nm (wavelength, 2 μm), followed by 35-nm Al film deposition via e-beam evaporation and lift-off process. The Ni (20)/Ti (15) stacks for the absorption measurement and the Pt (4)/Ni (14)/Ti (2) stacks for the spin pumping measurement are prepared by photolithography, e-beam evaporation, and lift-off process. A detailed layout and the transmission spectra are given in section S3.

### SAW measurements

The transmission S_12_ and S_21_ are measured using a vector network analyzer with an input power *P*_0_ = 10 mW (10 dBm) and a time-gating technique. The SAW transmission power *P*, *P*_±*k*_, and the absorption power Δ*P*_±*k*_ are given by *P* = *P*_0_ S_12_(600 mT)^2^ = *P*_0_ S_21_(600 mT)^2^, Δ*P*_+*k*_ (*H*) = *P* − *P*_+*k*_ *= P*_0_ S_12_(600 mT)^2^ − *P*_0_ S_12_(*H*)^2^, and Δ*P*_−*k*_ (*H*) = *P* − *P*_−*k*_ = *P*_0_ S_21_(600 mT)^2^ − *P*_0_ S_21_(*H*)^2^. The spin pumping experiment was performed by sending a radio frequency power of 20 dBm at the SAW center frequency with a signal generator and measuring the DC voltage with a Nanovoltmeter.

## Supplementary Materials

### This PDF file includes:

Supplementary Text Figs. S1 to S7 Legends for movies S1 to S4 References
