# Supplementary material for: Nonreciprocal magnetoacoustic waves with out-of-plane phononic angular momenta
Source: Sci Adv. 2024 Jul 10;10(28):eado2504. doi: 10.1126/sciadv.ado2504 (PMC11235162; doi:10.1126/sciadv.ado2504)
Supplement: Supplementary file 1 — Supplementary Text Figs. S1 to S7 Legends for movies S1 to S4 References [file sciadv.ado2504_sm.pdf]

Science Advances
AAAS

Supplementary Materials for

Nonreciprocal magnetoacoustic waves with out-of-plane phononic angular momenta

Liyang Liao *et al.*

Corresponding author: Wei Luo, hustluowei@gmail.com; Yoshichika Otani, yotani@issp.u-tokyo.ac.jp

Sci. Adv. **10**, eado2504 (2024)
DOI: 10.1126/sciadv.ado2504

The PDF file includes:

Supplementary Text
Figs. S1 to S7
Legends for movies S1 to S4
References

Other Supplementary Material for this manuscript includes the following:

Movies S1 to S4

1. Theory of SAW-driven FMR

In this section, we derive the theory of SAW-driven FMR from the magnetoelastic coupling between magnetization and strains. In a fully rotational symmetry magnet, which can approximately model the polycrystalline thin films deposited by evaporation, the magnetoelastic free energy reads (*18*, *43*)

$$F_{me} = b \sum_{i,j=x,y,z} \varepsilon_{ij} m_i m_j \text{ , (S1)}$$

where $\varepsilon_{ij} = (\partial_j u_i + \partial_i u_j)/2$ is the linear strain tensor defined on the displacement field $\boldsymbol{u} = (u_x, u_y, u_z)$, b is the magnetoelastic coupling constant, $\boldsymbol{m} = (m_x, m_y, m_z)$ is the normalized directional vector of the magnetization. An effective magnetic field is then generated(*12*, *16*)

$$\boldsymbol{h}^{\text{eff}} = -\frac{1}{\mu_0 M_S} \nabla F_{me} \text{, (S2)}$$

where μ_0 is the vacuum permeability. For FMR with small precession angle, the effective field can be calculated based on the ground state magnetization m_j.

To provide a simple insight on the polarized-phonon-induced FMR, we develop a two-dimensional (2D) model with 2D displacement (u_x, u_y) on a plane (x, y). The displacement and strains are continued at the $LiNbO_3$/Ni interface, and since the thickness of the magnetic film is much thinner than the wavelength, the displacement and strains in the magnetic layer can be approximated by the displacement and strains in the $LiNbO_3$ substrate. The small thickness also means that the interested area is close to the top surface, where the out-of-plane shear strain ε_{xz} and ε_{yz} should be zero, so that ε_{xz} and ε_{yz} are small in the Ni layer (*14*). Meanwhile ε_{yy} is zero in a plane wave, and ε_{zz} is usually small in $LiNbO_3$ (*44*). The remaining strains ε_{xx} and ε_{xy} are only related to the 2D displacement (u_x, u_y), allowing us to capture the essential physics in the SAW-driven FMR with our 2D model.

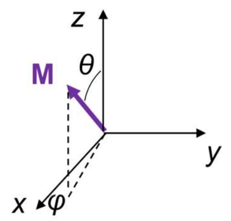

Fig. S1. Definition of angles characterizing the ground state magnetization orientation.

To account for the states with a tilted magnetization, we define a sphere coordinate system, with magnetization direction $\boldsymbol{m} = (\sin\theta\cos\varphi, \sin\theta\sin\varphi, \cos\theta)$ (Fig. S1). The $\boldsymbol{h}^{\text{eff}}$ components perpendicular to the ground state $\boldsymbol{m}$ are given by (*45*)

$$h_\theta^{\text{eff}} \equiv -\frac{1}{\mu_0 M_S}\frac{\partial F_{me}}{\partial \theta} = -\frac{b \sin 2\theta}{\mu_0 M_S}(\varepsilon_{xx}\cos^2\varphi + \varepsilon_{xy}\sin 2\varphi), \text{ (S3)}$$

$$h_\varphi^{\text{eff}} \equiv -\frac{1}{\mu_0 M_S \sin\theta}\frac{\partial F_{me}}{\partial \varphi} = \frac{b \sin\theta}{\mu_0 M_S}(\varepsilon_{xx}\sin 2\varphi - 2\varepsilon_{xy}\cos 2\varphi). \text{ (S4)}$$

As shown in Eq. (S3) and (S4), both effective field components vanish when $\theta = 0°$, or the magnetization is fully out-of-plane. Therefore, we need to rotate the magnetization from the z-axis.

The linearized Landau-Lifshitz-Gilbert equation takes the form (*43*, *46*, *47*)

$$\left\{F^{(2)}-\frac{iM_S}{\gamma}\begin{pmatrix}\alpha\omega & -\omega\\ \omega & \alpha\omega\end{pmatrix}\right\}\begin{pmatrix}m_\theta\\ m_\varphi\end{pmatrix}=\mu_0 M_S\begin{pmatrix}h_\theta^{\text{eff}}\\ h_\varphi^{\text{eff}}\end{pmatrix}, \text{(S5)}$$

where $(m_\theta, m_\varphi) = (\Delta\theta, \sin\theta\Delta\varphi)$ is the small derivation of $\boldsymbol{m}$ after Fourier transform in time, γ is the gyromagnetic ratio, α is the Gilbert damping, and $F^{(2)}$ is a self-adjoint two-by-two matrix, relating to the derivation of the magnetic part of the free energy density (modified from Eq. (6) in Ref.(*45*)). For the simplest case of static magnetic field H parallel to the ground state $\boldsymbol{m}$,

$$F^{(2)}=\mu_0 M_S H\begin{pmatrix}1 & 0\\ 0 & 1\end{pmatrix}. \text{(S6)}$$

Solving (Eq. S5) locally and keeping only the linear terms, one can obtain(*16*)

$$\begin{pmatrix}m_\theta\\ m_\varphi\end{pmatrix}=\frac{\gamma\mu_0}{\omega_F{}^2-\omega^2-i\alpha\omega_F\omega}\begin{pmatrix}(\omega_F-i\alpha\omega)h_\theta^{\text{eff}}-i\omega h_\varphi^{\text{eff}}\\ i\{\omega h_\theta^{\text{eff}}-i(\omega_F-i\alpha\omega)h_\varphi^{\text{eff}}\}\end{pmatrix}, \text{(S7)}$$

where $\omega_F = \gamma\mu_0 H$. To the leading order, the phonon absorption is given by the work per time per unit volume ΔP done by $\boldsymbol{h}^{\text{eff}}$ (*16*), i.e.

$$\begin{aligned}\Delta P &\equiv \text{Re}\left\{-i\omega\mu_0 M_S\left(m_\theta h_\theta^{\text{eff}*}+m_\varphi h_\varphi^{\text{eff}*}\right)\right\}\\ &=\alpha\omega^2\gamma\mu_0{}^2 M_S\frac{\omega^2\left(\left|h_\theta^{\text{eff}}\right|^2+\left|h_\varphi^{\text{eff}}\right|^2\right)-i\omega\omega_F\left(h_\varphi^{\text{eff}}h_\theta^{\text{eff}*}-h_\theta^{\text{eff}}h_\varphi^{\text{eff}*}\right)}{\left(\omega_F{}^2-\omega^2\right)^2+\alpha^2\omega_F{}^2\omega^2}, \quad \text{(S8)}\end{aligned}$$

where the star denotes complex conjugation. The second term, either positive or negative, depends on whether the polarization of the effective magnetic field is right- or left-handed circular. Since FMR has a right-hand circular polarization, the right-hand circularly polarized field couples twice as strongly as linear ones, and the left-hand circularly polarized field almost completely decouples. We denote this polarization dependent term ΔP_{chiral}, and replace the effective field with the strains using Eq. (S3)-(S4)

$$\Delta P_{\text{chiral}}=\frac{2\alpha\gamma b^2\omega^3\omega_F}{M_S}\frac{\text{Im}[\varepsilon_{xx}{}^*\varepsilon_{xy}]}{\left(\omega_F{}^2-\omega^2\right)^2+\alpha^2\omega_F{}^2\omega^2}\sin 2\theta\sin\theta(1+\cos 2\varphi). \text{(S9)}$$

It can be seen that ΔP_{chiral} is maximized when $\varphi = 0°$ or $180°$, which are the chosen experimental conditions in this work.

In a plane wave, the displacement field after Fourier transform in time is given by $(u_x, u_y) = (u_{x0}, u_{y0})e^{ikx}$, where u_{x0}, u_{y0} are complex constant amplitudes, and k is the wavevector of the SAW. Therefore

$$2\,\text{Im}[\varepsilon_{xx}{}^*\varepsilon_{xy}] = k^2\,\text{Im}[u_x{}^*u_y]. \text{ (S10)}$$

Using the relationship between the velocity field and the displacement field $v_i = -i\omega u_i$, we further get

$$k^2\,\text{Im}[u_x{}^*u_y]=\frac{k^2}{\omega^2}\text{Im}[v_x{}^*v_y]=\frac{k^2}{\omega}L_z, \text{ (S11)}$$

where L_z is given by Eq. (1) in the main text. Combining Eq. (S9)-(S11) and $\varphi = 0°$, we obtain Eq. (2) in the main text.

2. Angular momentum view for the nonreciprocity

We hereby use a semi-classical language to model the nonreciprocity, which more clearly shows the role of the angular momentum. As shown in Ref. (*18*), considering the predominance strains ε_{xx} and ε_{xy} only, by introducing $m_\pm = m_x \pm im_y$, and $\varepsilon_{x\pm} = \varepsilon_{xx} \pm 2i\varepsilon_{xy}$, the free energy of the magnetoelastic interaction Eq. (S1) can be rewritten as

$$F_{me} = \frac{b}{2} m_x (m_+ \varepsilon_{x-} + m_- \varepsilon_{x+}). \quad \text{(S12)}$$

Note that Ref. (*18*) defines the in-plane direction perpendicular to SAW propagation as the z axis and discusses an in-plane angular momentum along the same direction, while our z axis is defined along the film normal and we study the out-of-plane angular momentum, so that the above Eq. (S12) has a similar form as Eq. (3) in Ref. (*18*).

The free energy in Eq. (12) is also the Hamiltonian of the magnetoelastic interaction, showing that the angular momentum transfer can happen between the magnetic (spin) and the elastic (phonon) systems through the $S_\pm$ and the $\varepsilon_{x\pm}$ operators. In the electrical detection of such torque effect, one can analyze the Rayleigh wave-induced FMR in the spin-torque framework (*34*). Another experiment in YIG/GGG/YIG trilayer also demonstrated the angular momentum transfer between magnons and phonons via the magnetoelastic interaction (*48*).

We then write down the quantum operator related to phonons and their angular momenta via second quantization. The second quantization of the displacement vector, multiplied by the square root of mass(*49*), can be written as (SI Eq. (I.5) in Ref. (*18*))

$$\begin{aligned} \boldsymbol{u}(\boldsymbol{r}) &= \sum_{\boldsymbol{k},l} \sqrt{\frac{\hbar}{2m_a \omega_{\boldsymbol{k},l} N}} (\xi_{\boldsymbol{k}}^l a_{\boldsymbol{k}}^l e^{i(\boldsymbol{k}\cdot\boldsymbol{r}-\omega t)} + h.c.) \\ &= \sqrt{\frac{\hbar}{2m_a \omega N}} (\xi_+ a_+ e^{i(kx-\omega t)} + \xi_- a_- e^{i(kx-\omega t)} + h.c.) \quad \text{(S13)} \\ &+ \sum_{others} \sqrt{\frac{\hbar}{2m_a \omega_{\boldsymbol{k}',l'} N}} (\xi_{\boldsymbol{k}'}^{l'} a_{\boldsymbol{k}'}^{l'} e^{i(\boldsymbol{k}'\cdot\boldsymbol{r}-\omega_{\boldsymbol{k}',l'} t)} + h.c.), \end{aligned}$$

where $\xi_{\boldsymbol{k}}^l$ represents the polarization of the l-th phonon mode, m_a is the mass of an atom, with a wave vector $\boldsymbol{k}$. To study the SAW phonons circularly polarized in the XY plane, we take out the left-and right-handed circular polarized modes with $\xi_\pm = (1, \pm i, 0)/\sqrt{2}$, wave vector $\pm k\hat{x}$ and frequency ω. $a_{\boldsymbol{k}}^{l\dagger}$ and $a_{\boldsymbol{k}}^l$ creates and annihilates the general phonons with wave vector $\boldsymbol{k}$ in the l branch, and we focus on the given circularly polarized SAW phonons with creation and annihilation operators $a_\pm^\dagger$ and $a_\pm$, respectively. To describe the surface waves, Eq. (S13) is based on a two-dimensional plane with N unit cells in total. As we aim at showing the possibility of the angular momentum transfer effect between our SAW phonons and spins, we ignore the contribution from other modes in the following.

By some simple algebra like Eq. (S11), the angular momentum per mass given in Eq. (1) in the main text is microscopically equivalent to

$$\boldsymbol{L} = \sum_{\boldsymbol{r}} \boldsymbol{u}_r \times \dot{\boldsymbol{u}}_r \Big/ N, \quad \text{(S14)}$$

where $\boldsymbol{u}_r$ is the displacement vector of atom at $\boldsymbol{r}$. Eq. (S14) has a similar form as Eq. (1) by Q. Niu et al. (*49*), while $\boldsymbol{L}$ is an average value for one unit cell, and the displacement is not scaled

with the mass of the atom (like in Ref. (2)) in our definition. Instead, we only consider the long-wavelength phonons (μm scale), so the displacement is the same in one unit cell, allowing us to regard a unit cell as a single "atom" with mass m_a. Our expression thus represents the angular momentum per mass.

Replacing $\boldsymbol{u}(\boldsymbol{r})$ by Eq. (S13), and considering only the XY-circularly polarized SAW with wave vector $\pm k\hat{x}$ and frequency ω, one reach (*18*)

$$L_z = (a_+^\dagger a_+ - a_-^\dagger a_-)\hbar / m_a N \,. \quad \text{(S15)}$$

In other words, L_z is equivalent to the phonon number density difference between the right-and left-circularly polarized phonons. Since the strains are the derivations of displacements, $\varepsilon_{x\pm} = \varepsilon_{xx} \pm 2i\varepsilon_{xy} = \partial_x u_x \pm i(\partial_x u_y + \partial_y u_x)$, the operators for $\varepsilon_{x\pm}$ are (*18*)

$$\varepsilon_{x+} = -ik\sqrt{\frac{\hbar}{2m_a\omega N}}\left(a_+^\dagger e^{-i(kx-\omega t)} + a_- e^{-i(kx+\omega t)}\right),$$
$$\varepsilon_{x-} = ik\sqrt{\frac{\hbar}{2m_a\omega N}}\left(a_-^\dagger e^{i(kx+\omega t)} + a_+ e^{i(kx-\omega t)}\right). \quad \text{(S16)}$$

As shown in SI Eq. (I.9)-(I.12) in (*18*), these operators raises or lowers the quantum number of the circularly polarized phonon modes, with angular momentum change $\hbar$ in each process.

To describe the phonon-magnon conversion process, we next rewrite Eq. (S12) with magnon creation and annihilation operators. The equilibrium direction of the magnetization $\boldsymbol{m}$ is tilted by θ from the z axis, requiring a rotation for the coordinate system (Fig. S2). We then rewrite the magnetization in the coordinate system xyz as $\boldsymbol{m} = (m_x, m_y, m_z) = -(m_{x'}\cos\theta + m_{z'}\sin\theta, m_{y'}, -m_{x'}\sin\theta + m_{z'}\cos\theta)$. Here, $\boldsymbol{m'} = (m_{x'}, m_{y'}, m_{z'})$ is the magnetization in the coordinate system $x'y'z'$, which is rotated by θ around the y axis from the coordinate system xyz, and reversed all the axes. The reversion aims at orientating z' along the direction of the spin angular momentum $\boldsymbol{S}$, which is opposed to $\boldsymbol{m}$ due to the negative charge of electrons.

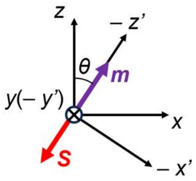

Fig. S2. **Rotation and inversion of the coordinate system**. The coordinate system xyz is transformed to coordinate system $x'y'z'$.

The well-known Holstein-Primakoff transformation (*50*) for magnons gives

$$S_{x'}(\boldsymbol{r}) = \frac{\sqrt{2S}}{2}(b_r + b_r^\dagger),$$
$$S_{y'}(\boldsymbol{r}) = \frac{\sqrt{2S}}{2i}(b_r - b_r^\dagger), \quad \text{(S17)}$$
$$S_{z'}(\boldsymbol{r}) = S - b_r^\dagger b_r,$$

where $(S_{x'}(\boldsymbol{r}), S_{y'}(\boldsymbol{r}), S_{z'}(\boldsymbol{r}))$ is the magnetic spin at $\boldsymbol{r}$, with spin number S, $b_r^\dagger$ and b_r are the creation and annihilation operators of magnon on site i, respectively. When S is large, there is $\boldsymbol{m'} \approx -(S_{x'}, S_{y'}, S_{z'})/S$. To analyze the magnons with finite wavevectors, the Fourier transformation provides

$$b_{\boldsymbol{r}}^{\dagger} = \sum_{\boldsymbol{k}''} e^{-i(\boldsymbol{k}''\cdot\boldsymbol{r}-\omega_{k''}t)} b_{\boldsymbol{k}''}^{\dagger} / \sqrt{N}, b_{\boldsymbol{r}} = \sum_{\boldsymbol{k}''} e^{i(\boldsymbol{k}''\cdot\boldsymbol{r}-\omega_{k''}t)} b_{\boldsymbol{k}''} / \sqrt{N}. \text{(S18)}$$

Combining the above expressions, we are now able to take out the contribution of hybridizing magnons and phonons with frequency ω and wavevector $\pm k\hat{x}$ in the total magnetoelastic interaction $\sum_{\boldsymbol{r}} F_{me}(\boldsymbol{r})$. We reach

$$F_{me}^{(k,\omega)} = \frac{ikb}{4}\sqrt{\frac{\hbar S}{m_a \omega}}\left\{a_+ b_k^{\dagger}(2\cos\theta - 1)\sin\theta + a\_b_{-k}^{\dagger}(2\cos\theta+1)\sin\theta\right\} + h.c. \text{ (S19)}$$

Note that we simplify (S19) by keeping only the particle number conserving two-particle processes and ignoring all the higher order processes, or the particle number non-conserving processes, as we are working in the linear regime. Also, these particle number conserving two-particle processes survive after averaging for one period ($2\pi/\omega$), which cancels out most of the irrelevant processes.

The $a_+ b_k^{\dagger}$ and $a\_b_{-k}^{\dagger}$ terms convert phonons into magnons, or convert phononic angular momentum (Eq. (S15)) into magnonic angular momentum. Applying the Fermi Golden rule, the phonon to magnon scatter rate can be estimated through the modulus square of the matrix elements, i.e.,

$$\left|F_{me}^{\pm}\right|^2 = \left|ikb/4\sqrt{\hbar S/m_a\omega}\right|^2 (2\cos\theta \pm 1)^2 \sin^2\theta. \text{ (S20)}$$

As both phonons and magnons carry angular momenta, the scatter rate is also associated with the angular momentum transfer rate. Considering the difference between the scatter rate from the phonons with oppose angular momentum, its θ dependence will be given by

$$(2\cos\theta+1)^2\sin^2\theta - (2\cos\theta-1)^2\sin^2\theta = 4\sin 2\theta \sin\theta, \text{(S21)}$$

which is the same as derived from the effective field method in S1, displayed in Eq. (2) in the main text.

Hence, the SAW nonreciprocity is associated with the difference of the angular momentum transfer rate between the phonons and the magnons, as shown in Fig. S3: With magnetization along $-z'$ and spin along z', when phonons with upward angular momenta are absorbed (a_+), there is $\boldsymbol{L}\cdot\boldsymbol{S} < 0$ and it tends to reduce the spin angular momentum, i.e., creating magnons efficiency (Fig. S3A). For phonons with downward angular momenta ($a\_$), there is $\boldsymbol{L}\cdot\boldsymbol{S} > 0$. Although $\boldsymbol{L}$ and $\boldsymbol{S}$ are not fully antiparallel, and it is still possible to create magnons, the scattering efficiency is smaller (Fig. S3B). The efficiency difference is described by the difference in the modulus square of the matrix elements $\left|F_{me}^{\pm}\right|^2$, as given in Eq. (S21), matching with the effective field helicity mismatch calculation Eq. (S9) and the experimental results.

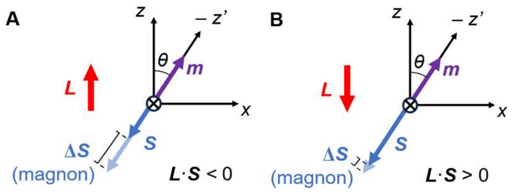

Fig. S3. The angular momentum view for the magnon-phonon coupling. The phonon-to-magnon scatter rate are different when $\boldsymbol{L}\cdot\boldsymbol{S} < 0$ (**A**) and $\boldsymbol{L}\cdot\boldsymbol{S} > 0$ (**B**). Note the $\boldsymbol{S}$ fluctuation amplitude $\Delta\boldsymbol{S}$ due to magnon generation is exaggerated for a clear view.

3. Sample layout and transmission spectrum

Figure S4A shows the detail of the geometry of the SAW delay line device for the absorption measurement. The separation between port 1 and port 2 IDTs is 300 μm. The IDTs have a width 400 nm and separation 600 nm (wavelength 2 μm). The device used for spin pumping measurement is shown in Fig. S4B. The Pt/Ni heterostructure is patterned into a rectangle with dimension 100 μm × 60 μm. Figure S4C displays the transmission spectrum at zero magnetic field in the $\phi_k = 150°$ device for absorption measurement. The magnetic field-dependence data in the main text is collected at the center frequency 1.785 GHz labeled by the black arrow. Center frequency for other devices is taken in a similar way.

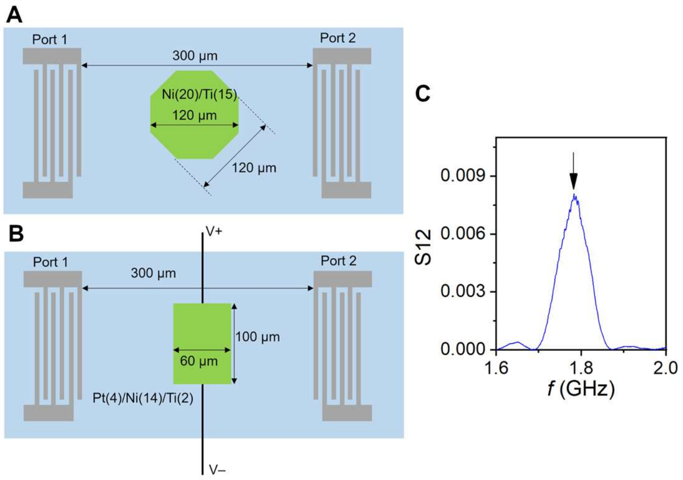

Fig. S4. Geometry of the SAW devices. (**A**) Geometry of the SAW delay line device for the absorption measurement. (**B**) Geometry of the SAW delay line device for the spin pumping measurement. (**C**) Transmission spectrum of the SAW delay line device for the absorption measurement with $\phi_k = 150°$.

4. Calculation of SAW-driven FMR in Ni film

The magnetization tilting angle θ is calculated using a macrospin model. The magnetic part of the free energy density is

$$F_m = -\mu_0 M_S H(\cos\theta_H \cos\theta + \sin\theta_H \sin\theta\cos\varphi) + K_{an}\cos^2\theta \,, \quad \text{(S22)}$$

where H is the applied field, θ_H is the magnetic field tilting angle in the xz plane, φ is the in-plane angle of the magnetization, the saturated magnetization $M_S \approx 2.8\times10^5$ A/m for Ni (*51*), and anisotropy $K_{an} \approx \mu_0 M_S^2/2$ mainly arising from the demagnetization field. For a given magnetic field H along direction θ_H, the magnetic ground state can be calculated by minimizing F_m and getting the corresponding θ, with φ always equals to 0°.

The FMR frequency is then calculated from the Landau-Lifshitz equation (*52*). For the present consideration, one may drop the damping and effective fields from Eq. (S5) to obtain (*45*)

$$iM_S\omega_F \begin{pmatrix} m_\theta \\ m_\varphi \end{pmatrix} = \gamma \begin{pmatrix} 0 & \dfrac{1}{\sin^2\theta}\dfrac{\partial^2 F_m}{\partial\varphi^2} \\ -\dfrac{\partial^2 F_m}{\partial\theta^2} & 0 \end{pmatrix} \begin{pmatrix} m_\theta \\ m_\varphi \end{pmatrix}, \quad \text{(S23)}$$

where ω_F is the angular frequency of the mode, and the diagonal terms are zero because $\partial^2 F_m / \partial\theta\partial\varphi = \partial^2 F_m / \partial\varphi\partial\theta = 0$ for $\varphi = 0°$. Taking $\gamma/2\pi$ = 28GHz/T, diagonalizing Eq. (S23) with the θ given by minimizing Eq. (S22), we get the FMR eigenfrequency ω_F, which is a function of H and θ_H. With H = 300 mT along θ_H = 3°, which is the resonance condition in Fig. 2B, Eq. (S23) gives and resonance frequency $\omega_F \approx$ 1.8 GHz and $\theta \approx$ 37°. More comprehensive discussion about H, θ_H and θ, ω_F can be found in Ref. (*44*, *51*).

Now based on the calculated θ and ω_F, we can calculate the nonreciprocal power given by Eq. (2), which is used for plotting Fig. 2E. The replacement of ω_F is based on an approximation that the Ni magnetization under the resonance condition can be modeled by the paramagnetic moments under the same resonance condition and with the same tilting angle θ. We take the excitation frequency ω as the SAW frequency, and the damping constant α = 0.35 for the calculation. The relatively large damping parameter might be caused by the inhomogeneous broadening at low frequency(*53*, *54*). Note that the plot in Fig. 2E is in arbitrary unit and does not contain the information of the magnitude of L_z and the magnetoelastic constant b, as we are mainly interested in the evolution of the nonreciprocal absorption with the changing direction and magnitude of the magnetic field.

5. COMSOL simulation

In COMSOL simulation, we start with the single period simulation with periodic boundary condition, allowing us to analyze the SAW properties in a small unit cell. The unit cell is shown in Fig. S5A, with in-plane dimensions equal to the wavelength $\lambda = 2$ μm, and thickness equals to $6\lambda = 12$ μm. A perfect matching layer with thickness 2 μm is set at the bottom to reduce the reflection from the bottom. The mass of the metallic IDTs is ignored. Due to the periodicity, the SAW is standing wave in this simulation.

We first show the admittance as a function of frequency in devices with different orientation in Fig. S5B to find the resonance frequency. The peaks with largest admittance characterize the resonance frequencies 1.827 GHz, 1.781 GHz, 1.835 GHz and 1.937 GHz for $\phi_k = 0°$, 30°, 60° and 90°, respectively. The properties at 120° and 150° are the same as that at 60° and 30°, respectively.

Then we map the strain distributions in these devices. At $\phi_k = 0°$ (Fig. S5C), only ε_{xx} exists and ε_{xy} is zero in the whole device, showing that the transverse and longitudinal modes are decoupled, with only the longitudinal mode couples to the piezoelectricity. At $\phi_k = 30°$ or 150° (Fig. S5D), a sizable ε_{xy} appears, whose distribution shifts from ε_{xx} for approximately a quarter wavelength. Sizable ε_{xy} with distribution shifts from ε_{xx} also appears along $\phi_k = 60°$ or 120° (Fig. S5E), although the shift is smaller than a quarter wavelength. At both 30° and 60°, ε_{xy} shifts rightward compared with ε_{xx}. When ϕ_k rotates to 90°, a very small ε_{xy} exist, which shifts leftward compared with ε_{xx}. Our results are consistent with a recent magnetoacoustics absorption report (*55*).

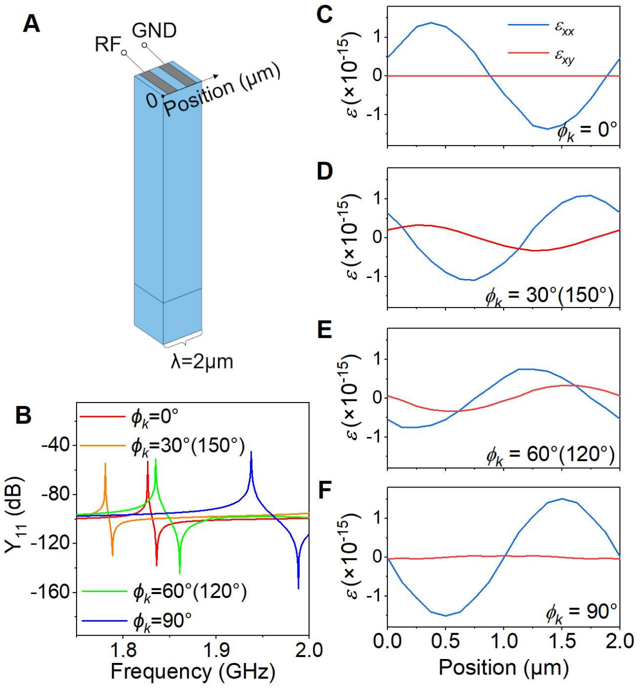

Fig. S5. Single-wavelength COMSOL Simulation. (**A**) Schematic of the unit cell in the single period simulation. (**B**) Admittance as a function of frequency in devices with different orientation. (**C-F**) Strain distributions in devices with different orientation.

We then expect large circular polarizations in the propagating waves along ϕ_k = 30° (150°) and 60° (120°), while 30° (150°) allows a slightly larger circular polarization. Along ϕ_k = 0°, no circular polarization can exist. A small circular polarization with opposite sign compared to 30° (150°) and 60° (120°) is expected along ϕ_k = 90°. These correspondence origins from the fact that standing wave is the superposition of propagating waves opposite direction, $(u_x, u_y)_S = (u_{x0}, u_{y0})(e^{ikx} + e^{-ikx})$. The distribution of ε_{xx} and ε_{xy} in the sanding wave are given by $\mathrm{Re}[iku_{x,S}]$ and $\mathrm{Re}[iku_{y,S}]/2$. It is easy to see that the phase difference between u_{x0} and u_{y0}, which determines the circular polarization of the propagating waves, is given by the distribution shift of ε_{xx} and ε_{xy} in the sanding wave. The amplitude of u_{x0} and u_{y0}, are given by the amplitude of ε_{xx} and ε_{xy} in the sanding wave. Hence, the angular momentum carried by the propagating waves can be obtained by analyzing the standing waves showing in Fig. S5C-F.

We show the emission of the angular-momentum-carrying waves along ϕ_k = 150° using a larger device structure shown in Fig. 3C. 9 IDT fingers are used, with 5 fingers for RF signal and 4 fingers for ground. The wavelength is 2 μm, corresponding to IDT fingers with width 500 nm and separation 500 nm. The z component of the angular momentum in (Eq.1) is calculated as a function of time at the surface of the substrate. The integrated L_z in the AB region and the CD region are used to reflect the angular momentum carried by the + k and – k SAWs, respectively. The simulation along ϕ_k = 0° is done in a similar way.

We next add the Ni(20)/Ti(15) layers onto the transport part in the above device (Fig S6A) and analyze the strain distribution. In Fig. S6, we show the time-evolution of the strain components in the Ni layer near the $LiNbO_3$ substrate, which is representative for analyzing the magnetoelastic coupling. The time evolutions of the strains in the whole structure are attached in the supplementary animations. As shown in Fig. S6B, the strains ε_{xz}, ε_{yz} and ε_{yy} are negligible. The ε_{yy} vanishes as the SAW studied here is a plane wave, and the ε_{xz}, ε_{yz} are limited by the film thickness (35nm) which is much smaller than the wavelength (2 μm). In addition to the ε_{xx} and ε_{xy} we have analyzed in the two-dimensional model, a finite ε_{zz} appears in the simulation. Taking ε_{zz} into account, Eq. (S3) would be modified to

$$h_\theta^{\mathrm{eff}} \equiv -\frac{1}{\mu_0 M_S}\frac{\partial F_{me}}{\partial \theta} = -\frac{b\sin 2\theta}{\mu_0 M_S}(\varepsilon_{xx}\cos^2\varphi - \varepsilon_{zz} + \varepsilon_{xy}\sin 2\varphi), \quad \text{(S24)}$$

with (S4) remains the same. When φ = 0°, (S24) is equivalent to replacing ε_{xx} in Eq. (S3) with a renormalized $\varepsilon_{xx}' = \varepsilon_{xx} - \varepsilon_{zz}$, and all other following analysis would not be changed.

To evaluate the decay length in the depth direction and the propagation loss, we calculated the acoustic energy flow in the substrate with different SAW propagation direction. As shown in the results (Fig. S6C), while all the SAWs have a decay length comparable to the wavelength in the depth direction, only SAWs with ϕ_k = 0°(180°) and 90° have good energy confinement at the surface. When ϕ_k = 30°(150°) or 60°(120°), finite leakage waves propagating into the substrate appears, increasing the propagation loss in these directions.

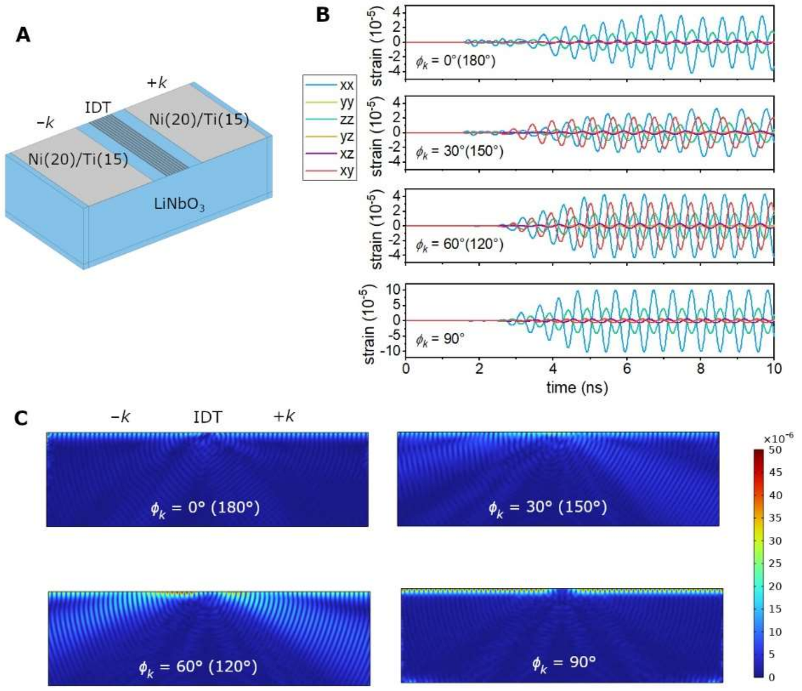

Fig. S6. COMSOL Simulation for SAW propagation. (**A**) Schematic of COMSOL simulation setup. (**B**) Time-evolution of all strain components in the Ni layer close to the $LiNbO_3$ substrate, in devices different SAW propagation direction. (**C**) Acoustic energy flows in the $LiNbO_3$ substrate in devices with different SAW propagation direction.

Supplementary Movie 1. Strain distribution along the device depth for SAW propagating along the 0° in-plane crystal direction. The horizontal axis represents the device depth direction (-z direction), i.e., 0 nm is the top surface, 15 nm is the Ni/Ti interface, and 35nm is the Ni/$LiNbO_3$ interface. The movie shows the time evolution of each strain component.

Supplementary Movie 2. Strain distribution along the device depth for SAW propagating along the 30° (150°) in-plane crystal direction.

Supplementary Movie 3. Strain distribution along the device depth for SAW propagating along the 60° (120°) in-plane crystal direction.

Supplementary Movie 4. Strain distribution along the device depth for SAW propagating along the 90° in-plane crystal direction.

6. Piezoelectric effect and phonon circular polarization

We now provide a parsed approximation for a straightforward understanding on how the phononic circular polarization emerges from the piezoelectric effect. The coupling between the displacement components and electric potential in SAWs can be described by the Christoffel equation (*56–58*). For a plane SAW at the surface of a piezoelectric material, with propagation direction along x-axis and surface normal along z-axis (Fig. S7A), the Christoffel equation reads

$$\begin{pmatrix} \Gamma_{11}-\rho v_s^2 & \Gamma_{12} & \Gamma_{13} & \Gamma_{14} \\ \Gamma_{12} & \Gamma_{22}-\rho v_s^2 & \Gamma_{23} & \Gamma_{24} \\ \Gamma_{13} & \Gamma_{23} & \Gamma_{33}-\rho v_s^2 & \Gamma_{34} \\ \Gamma_{14} & \Gamma_{24} & \Gamma_{34} & \Gamma_{44} \end{pmatrix} \begin{pmatrix} A_x \\ A_y \\ A_z \\ A_U \end{pmatrix} = 0, \text{(S25)}$$

where ρ is the mass density of the piezoelectric material, v_s is the velocity of the SAW, Γ_{ij} parameters are related to the elastic, piezoelectric and permittivity matrices, and $(A_x, A_y, A_z, A_U)^T$ describes the wavefunction of the SAW. The displacement and electric potential are given by

$$u_i(x,z) = A_i \exp[-i\omega(\alpha z + x)/v_s], \text{(S26)}$$

$$U(x,z) = A_U \exp[-i\omega(\alpha z + x)/v_s], \text{(S27)}$$

where ω is the circular frequency of the SAW, α is the decay factor along the depth direction ($-z$ direction) of the material. For a surface wave decaying along the depth direction, the imaginary part of α should be positive. The Γ_{ij} parameters are also related to α as a result of the inhomogeneous distribution of the displacement and the potential. Hence, Eq. (S25) is an equation about α and v_s, and is usually solved in a self-consistent manner, with complex selection rules to distinguish SAW and leaky waves (*56*, *59*). Here, to give a qualitative explanation on the phononic angular momentum, we skip this analysis and simply assume $\alpha = i$, or the studied wave has a delay length equals to the wavelength.

The coupling between electric potential and the displacements, as shown in Fig. 3F, is governed by Γ_{i4} (i = 1, 2, 3), which are determined by the piezoelectric matrix and α

$$\Gamma_{14} = e_{35}\alpha^2 + \alpha(e_{15}+e_{31}) + e_{11},$$
$$\Gamma_{24} = e_{34}\alpha^2 + \alpha(e_{14}+e_{36}) + e_{16}, \text{(S28)}$$
$$\Gamma_{34} = e_{33}\alpha^2 + \alpha(e_{13}+e_{35}) + e_{15},$$

where e_{ij} are the matrix elements of the piezoelectric matrix $[e]$. In the 3×6 matrix $[e]$, i = 1, 2, 3 correspond to the electric field components E_x, E_y, E_z, respectively, and j = 1, 2, 3, 4, 5, 6 correspond to the strains ε_{xx}, ε_{yy}, ε_{zz}, ε_{yz}, ε_{zx}, ε_{xy}, respectively.

The complex α brings about imaginary parts in Γ_{i4} in Eq. (S28). We then consider a mixing piezoelectric parameter $\Gamma_{\text{mix}} = 2\text{Im}[\Gamma_{14}^*\Gamma_{24}]$, and a normalized $\Gamma_{\text{mix}} = 2\text{Im}[\Gamma_{14}^*\Gamma_{24}]/(|\Gamma_{14}|^2 + |\Gamma_{24}|^2)$. The relationship between these two parameters and the out-of-plane phononic angular momentum can be understood by considering the following process: when u_x, u_y are excited by a periodic potential U, their magnitude can be estimated by $u_x \sim \Gamma_{14}U$, $u_y \sim \Gamma_{24}U$. Then, the phononic angular momentum ~ $\text{Im}[u_x^* u_y]$ ~ $\text{Im}[\Gamma_{14}^*\Gamma_{24}]$. To estimate the strength of the circular polarization, we can consider a normalized angular momentum ~ $2\text{Im}[u_x^* u_y]/(|u_x|^2 + |u_y|^2)$ ~ $2\text{Im}[\Gamma_{14}^*\Gamma_{24}]/(|\Gamma_{14}|^2 + |\Gamma_{24}|^2)$. Therefore, Γ_{mix} can be used to estimate the strength of the piezoelectric mixing effect that generating the out-of-plane phononic angular momentum, and the normalized Γ_{mix} can be used to estimate the strength of the circular polarization.

By rotating the piezoelectric matrix [e] of $LiNbO_3$ (*60*) using the standard Euler and Bond transform matrices (*58*, *60*), we can obtain [e] for the 128° Y-cut $LiNbO_3$ substrate with x-axis along a given direction labeled by ϕ_k. Fig. S7B plots the corresponding Γ_{mix} and normalized Γ_{mix} as functions of ϕ_k on the 128° Y-cut $LiNbO_3$. It can be seen that the Γ_{mix} curve can qualitatively reproduce the tendency of the experimental ΔP_n curve in Fig. 3B. The data between 180° and 360° is simply the opposite of the data between 0° and 180°, as displayed in Fig. 1. For most of ϕ_k, Γ_{mix} is nonzero, and large Γ_{mix} appears at ϕ_k between X and Y' axes. At $\phi_k = 0°$ (Y' axis), $\Gamma_{mix} = 0$ because $\Gamma_{24} = 0$, i.e., the transverse deformation u_y is decoupled with the electric field and the longitudinal deformation. The normalized Γ_{mix} has a maximum value ~ 0.5, consistent with the large circular polarization reflected by the large nonreciprocity in the experiment. The parameter Γ_{mix} based on Eq. (S28) also shows a zero value for the Y-cut z-propagating SAW, consistent with a previous study reporting no nonreciprocity (*44*). Though, the estimation here is simplified by skipping the complicated solving process of the Christoffel equation, aiming at clarifying the existence of the mechanism shown in Fig. 3F, and a quantitative agreement with the experiment shall not be expected based on this analysis.

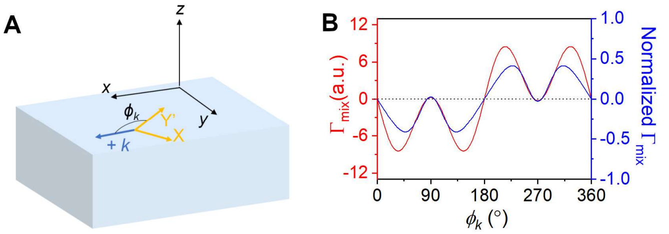

Fig. S7. Piezoelectric strain-mixing effect. (**A**) Schematic of the coordinate system for the Christoffel equation of the SAW. (**B**) Propagation direction dependence of the mixing piezoelectric parameters.

REFERENCES AND NOTES

1. D. MacNeill, G. M. Stiehl, M. H. D. Guimaraes, R. A. Buhrman, J. Park, D. C. Ralph, Control of spin-orbit torques through crystal symmetry in WTe_2/ferromagnet bilayers. *Nat. Phys.* **13**, 300–305 (2017).

2. L. Liu, C. Zhou, X. Shu, C. Li, T. Zhao, W. Lin, J. Deng, Q. Xie, S. Chen, J. Zhou, R. Guo, H. Wang, J. Yu, S. Shi, P. Yang, S. Pennycook, A. Manchon, J. Chen, Symmetry-dependent field-free switching of perpendicular magnetization. *Nat. Nanotechnol.* **16**, 277–282 (2021).

3. M. Kimata, H. Chen, K. Kondou, S. Sugimoto, P. K. Muduli, M. Ikhlas, Y. Omori, T. Tomita, A. H. MacDonald, S. Nakatsuji, Y. Otani, Magnetic and magnetic inverse spin Hall effects in a non-collinear antiferromagnet. *Nature* **565**, 627–630 (2019).

4. X. Chen, S. Shi, G. Shi, X. Fan, C. Song, X. Zhou, H. Bai, L. Liao, Y. Zhou, H. Zhang, A. Li, Y. Chen, X. Han, S. Jiang, Z. Zhu, H. Wu, X. Wang, D. Xue, H. Yang, F. Pan, Observation of the antiferromagnetic spin Hall effect. *Nat. Mater.* **20**, 800–804 (2021).

5. L. Wang, J. Xiong, B. Cheng, Y. Dai, F. Wang, C. Pan, T. Cao, X. Liu, P. Wang, M. Chen, S. Yan, Z. Liu, J. Xiao, X. Xu, Z. Wang, Y. Shi, S.-W. Cheong, H. Zhang, S.-J. Liang, F. Miao, Cascadable in-memory computing based on symmetric writing and readout. *Sci. Adv.* **8**, eabq6833 (2022).

6. C. Yun, H. Guo, Z. Lin, L. Peng, Z. Liang, M. Meng, B. Zhang, Z. Zhao, L. Wang, Y. Ma, Y. Liu, W. Li, S. Ning, Y. Hou, J. Yang, Z. Luo, Efficient current-induced spin torques and field-free magnetization switching in a room-temperature van der Waals magnet. *Sci. Adv.* **9**, eadj3955 (2023).

7. F. Wang, G. Shi, K.-W. Kim, H.-J. Park, J. G. Jang, H. R. Tan, M. Lin, Y. Liu, T. Kim, D. Yang, S. Zhao, K. Lee, S. Yang, A. Soumyanarayanan, K.-J. Lee, H. Yang, Field-free switching of perpendicular magnetization by two-dimensional $PtTe_2$/WTe_2 van der Waals heterostructures with high spin Hall conductivity. *Nat. Mater.* **23**, 768–774, (2024).

8. Y. Liu, G. Shi, D. Kumar, T. Kim, S. Shi, D. Yang, J. Zhang, C. Zhang, F. Wang, S. Yang, Y. Pu, P. Yu, K. Cai, H. Yang, Field-free switching of perpendicular magnetization at room temperature using out-of-plane spins from $TaIrTe_4$. *Nat. Electron.* **6**, 732–738 (2023).

9. J. Puebla, Y. Hwang, K. Kondou, Y. Otani, Progress in spinconversion and its connection with band crossing. *Ann. Phys.* **534**, 2100398 (2022).

10. M. Weiler, H. Huebl, F. S. Goerg, F. D. Czeschka, R. Gross, S. T. B. Goennenwein, Spin pumping with coherent elastic waves. *Phys. Rev. Lett.* **108**, 176601 (2012).

11. M. Xu, J. Puebla, F. Auvray, B. Rana, K. Kondou, Y. Otani, Inverse Edelstein effect induced by magnon-phonon coupling. *Phys. Rev. B* **97**, 180301 (2018).

12. M. Weiler, L. Dreher, C. Heeg, H. Huebl, R. Gross, M. S. Brandt, S. T. B. Goennenwein, Elastically driven ferromagnetic resonance in nickel thin films. *Phys. Rev. Lett.* **106**, 117601 (2011).

13. M. Kü, M. Heigl, L. Flacke, A. Hörner, M. Weiler, M. Albrecht, A. Wixforth, Nonreciprocal Dzyaloshinskii-Moriya magnetoacoustic waves. *Phys. Rev. Lett.* **125**, 217203 (2020).

14. S. Tateno, Y. Nozaki, Y. Nozaki, Highly nonreciprocal spin waves excited by magnetoelastic coupling in a Ni/Si bilayer. *Phys. Rev. Appl.* **13**, 034074 (2020).

15. R. Sasaki, Y. Nii, Y. Iguchi, Y. Onose, Nonreciprocal propagation of surface acoustic wave in Ni/LiNbO3. *Phys. Rev. B* **95**, 020407 (2017).

16. M. Xu, K. Yamamoto, J. Puebla, K. Baumgaertl, B. Rana, K. Miura, H. Takahashi, D. Grundler, S. Maekawa, Y. Otani, Nonreciprocal surface acoustic wave propagation via magneto-rotation coupling. *Sci. Adv.* **6**, eabb1724 (2020).

17. P. J. Shah, D. A. Bas, I. Lisenkov, A. Matyushov, N. X. Sun, M. R. Page, Giant nonreciprocity of surface acoustic waves enabled by the magnetoelastic interaction. *Sci. Adv.* **6**, eabc5648 (2020).

18. R. Sasaki, Y. Nii, Y. Onose, Magnetization control by angular momentum transfer from surface acoustic wave to ferromagnetic spin moments. *Nat. Commun.* **12**, 2599 (2021).

19. L. Thevenard, I. S. Camara, S. Majrab, M. Bernard, P. Rovillain, A. Lemaître, C. Gourdon, J. Y. Duquesne, Precessional magnetization switching by a surface acoustic wave. *Phys. Rev. B* **93**, 134430 (2016).

20. T. Kawada, M. Kawaguchi, T. Funato, H. Kohno, M. Hayashi, Acoustic spin Hall effect in strong spin-orbit metals. *Sci. Adv.* **7**, eabd9697 (2021).

21. T. Yokouchi, S. Sugimoto, B. Rana, S. Seki, N. Ogawa, S. Kasai, Y. Otani, Creation of magnetic skyrmions by surface acoustic waves. *Nat. Nanotechnol.* **15**, 361–366 (2020).

22. R. Chen, C. Chen, L. Han, P. Liu, R. Su, W. Zhu, Y. Zhou, F. Pan, C. Song, Ordered creation and motion of skyrmions with surface acoustic wave. *Nat. Commun.* **14**, 4427 (2023).

23. K. Y. Bliokh, Elastic spin and orbital angular momenta. *Phys. Rev. Lett.* **129**, 204303 (2022).

24. K. Y. Bliokh, F. Nori, Transverse spin and surface waves in acoustic metamaterials. *Phys. Rev. B* **99**, 020301 (2019).

25. L. Zhang, Q. Niu, Chiral phonons at high-symmetry points in monolayer hexagonal lattices. *Phys. Rev. Lett.* **115**, 115502 (2015).

26. H. Zhu, J. Yi, M. Y. Li, J. Xiao, L. Zhang, C. W. Yang, R. A. Kaindl, L. J. Li, Y. Wang, X. Zhang, Observation of chiral phonons. *Science* **359**, 579–582 (2018).

27. J. Kishine, A. S. Ovchinnikov, A. A. Tereshchenko, Chirality-induced phonon dispersion in a noncentrosymmetric micropolar crystal. *Phys. Rev. Lett.* **125**, 245302 (2020).

28. J. Luo, T. Lin, J. Zhang, X. Chen, E. R. Blackert, R. Xu, B. I. Yakobson, H. Zhu, Large effective magnetic fields from chiral phonons in rare-earth halides. *Science* **382**, 698–702 (2023).

29. Y. Liu, C. S. Lian, Y. Li, Y. Xu, W. Duan, Pseudospins and topological effects of phonons in a Kekulé lattice. *Phys. Rev. Lett.* **119**, 255901 (2017).

30. K. Kim, E. Vetter, L. Yan, C. Yang, Z. Wang, R. Sun, Y. Yang, A. H. Comstock, X. Li, J. Zhou, L. Zhang, W. You, D. Sun, J. Liu, Chiral-phonon-activated spin Seebeck effect. *Nat. Mater.* **22**, 322–328 (2023).

31. J. Im, C. H. Kim, H. Jin, Ferroelectricity-driven phonon berry curvature and nonlinear phonon Hall transports. *Nano Lett.* **22**, 8281–8286 (2022).

32. T. Yu, Y. M. Blanter, G. E. W. Bauer, Chiral pumping of spin waves. *Phys. Rev. Lett.* **123**, 247202 (2019).

33. W. Fu, Z. Shen, Y. Xu, C. L. Zou, R. Cheng, X. Han, H. X. Tang, Phononic integrated circuitry and spin–orbit interaction of phonons. *Nat. Commun.* **10**, 2743 (2019).

34. C. Chen, L. Han, P. Liu, Y. Zhang, S. Liang, Y. Zhou, W. Zhu, S. Fu, F. Pan, C. Song, Direct-current electrical detection of surface-acoustic-wave-driven ferromagnetic resonance. *Adv. Mater.* **35**, e2302454 (2023).

35. Y. Hwang, J. Puebla, K. Kondou, Y. Otani, Voltage signals caused by surface acoustic wave driven ferromagnetic resonance under out-of-plane external fields. *Adv. Mater. Interfaces* **9**, 2201432 (2022).

36. M. Hu, F. Li Duan, Design, fabrication and characterization of SAW devices on LiNbO3 bulk and ZnO thin film substrates. *Solid State Electron.* **150**, 28–34 (2018).

37. K. Kondou, H. Sukegawa, S. Kasai, S. Mitani, Y. Niimi, Y. Otani, Influence of inverse spin Hall effect in spin-torque ferromagnetic resonance measurements. *Appl. Phys. Express* **9**, 023002 (2016).

38. X. Fan, E. Himbeault, Y. S. Gui, A. Wirthmann, G. Williams, D. Xue, C.-M. Hu, Electrical detection of large cone angle spin precession from the linear to the nonlinear regime. *J. Appl. Phys.* **108**, 046102 (2010).

39. V. I. Anisimkin, Anisotropy of the acoustic plate modes in ST-quartz and 128°Y-$LiNbO_3$. *IEEE Trans. Ultrason. Ferroelectr. Freq. Control* **61**, 120–132 (2014).

40. Y. Chen, M. Kadic, S. Guenneau, M. Wegener, Isotropic chiral acoustic phonons in 3D quasicrystalline metamaterials. *Phys. Rev. Lett.* **124**, 235502 (2020).

41. M. DC, D.-F. Shao, V. D.-H. Hou, A. Vailionis, P. Quarterman, A. Habiboglu, M. B. Venuti, F. Xue, Y.-L. Huang, C.-M. Lee, M. Miura, B. Kirby, C. Bi, X. Li, Y. Deng, S.-J. Lin, W. Tsai, S. Eley, W.-G. Wang, J. A. Borchers, E. Y. Tsymbal, S. X. Wang, Observation of anti-damping spin–orbit torques generated by in-plane and out-of-plane spin polarizations in $MnPd_3$. *Nat. Mater.* **22**, 591–598 (2023).

42. J. Sun, S. Shi, J. Wang, Acoustic waves induced by Einstein–de Haas effect in the ultrafast core reversal of magnetic vortex. *Phys. Rev. Lett.* **130**, 256701 (2023).

43. T. P. Lyons, J. Puebla, K. Yamamoto, R. S. Deacon, Y. Hwang, K. Ishibashi, S. Maekawa, Y. Otani, Acoustically driven magnon-phonon coupling in a layered antiferromagnet. *Phys. Rev. Lett.* **131**, 196701 (2023).

44. L. Dreher, M. Weiler, M. Pernpeintner, H. Huebl, R. Gross, M. S. Brandt, S. T. B. Goennenwein, Surface acoustic wave driven ferromagnetic resonance in nickel thin films: Theory and experiment. *Phys. Rev. B* **86**, 134415 (2012).

45. K. Yamamoto, M. Xu, J. Puebla, Y. Otani, S. Maekawa, Interaction between surface acoustic waves and spin waves in a ferromagnetic thin film. *J. Magn. Magn. Mater.* **545**, 168672 (2022).

46. K. Yamamoto, W. Yu, T. Yu, J. Puebla, M. Xu, S. Maekawa, G. Bauer, Non-reciprocal pumping of surface acoustic waves by spin wave resonance. *J. Physical Soc. Japan* **89**, 113702 (2020).

47. Y. Hwang, J. Puebla, K. Kondou, C. Gonzalez-Ballestero, H. Isshiki, C. S. Muñoz, L. Liao, F. Chen, W. Luo, S. Maekawa, Y. Otani, Strongly coupled spin waves and surface acoustic waves at room temperature. *Phys. Rev. Lett.* **132**, 056704 (2024).

48. K. An, A. N. Litvinenko, R. Kohno, A. A. Fuad, V. V. Naletov, L. Vila, U. Ebels, G. De Loubens, H. Hurdequint, N. Beaulieu, J. Ben Youssef, N. Vukadinovic, G. E. W. Bauer, A. N. Slavin, V. S. Tiberkevich, O. Klein, Coherent long-range transfer of angular momentum between magnon Kittel modes by phonons. *Phys. Rev. B* **101**, 060407 (2020).

49. L. Zhang, Q. Niu, Angular momentum of phonons and the Einstein–de Haas effect. *Phys. Rev. Lett.* **112**, 085503 (2014).

50. G. Go, S. K. Kim, K.-J. Lee, Topological magnon-phonon hybrid excitations in two-dimensional ferromagnets with tunable Chern numbers. *Phys. Rev. Lett.* **123**, 237207 (2019).

51. L. Liao, J. Puebla, K. Yamamoto, J. Kim, S. Maekawa, Y. Hwang, Y. Ba, Y. Otani, Valley-selective phonon-magnon scattering in magnetoelastic superlattices. *Phys. Rev. Lett.* **131**, 176701 (2023).

52. D. D. Stancil, A. Prabhakar, *Spin Waves:Theory and Applications* (Springer, 2009).

53. T. Wimmer, M. Althammer, L. Liensberger, N. Vlietstra, S. Geprägs, M. Weiler, R. Gross, H. Huebl, Spin transport in a magnetic insulator with zero effective damping. *Phys. Rev. Lett.* **123**, 257201 (2019).

54. M. Collet, X. de Milly, O. d'Allivy Kelly, V. V. Naletov, R. Bernard, P. Bortolotti, J. Ben Youssef, V. E. Demidov, S. O. Demokritov, J. L. Prieto, M. Muñoz, V. Cros, A. Anane, G. de Loubens, O. Klein, Generation of coherent spin-wave modes in yttrium iron garnet microdiscs by spin-orbit torque. *Nat. Commun.* **7**, 10377 (2016).

55. R. Gao, J. Xue, H. Wu, Y. Ye, J. Wang, Q. Liu, Acoustically driven spin wave resonance in Ni/128° Y-cut $LiNbO_3$ hybrid devices with the beam steering effect. *Appl. Phys. Lett.* **123**, 232401 (2023).

56. K. Nakamura, M. Kazumi, H. Shimizu, "SH-type and Rayleigh-type surface waves on rotated Y-Cut LiTaO3," in *1977 Ultrasonics Symposium* (IEEE, 1977), pp. 819–822.

57. K. Hashimoto, *Surface Acoustic Wave Devices in Telecommunications* (Springer, 2000).

58. J. J. Campbell, W. R. Jones, A method for estimating optimal crystal cuts and propagation directions for excitation of piezoelectric surface waves. *IEEE Trans. Sonics Ultrason.* **15**, 209–217 (1968).

59. S. V Biryukov, M. Weihnacht, "The effective permittivity in the complex plane and a simple estimation method for leaky wave slowness," in *1996 IEEE Ultrasonics Symposium Proceedings* (IEEE, 1996), vol. 1, pp. 221–224.

60. W. Yue, J. Yi-jian, Crystal orientation dependence of piezoelectric properties in $LiNbO_3$ and $LiTaO_3$. *Opt. Mater. (Amsterdam)* **23**, 403–408 (2003).
