# Supplementary figures and images for: Nonreciprocal magnetoacoustic waves with out-of-plane phononic angular momenta
Source: Sci Adv. 2024 Jul 10;10(28):eado2504. doi: 10.1126/sciadv.ado2504 (PMC11235162; doi:10.1126/sciadv.ado2504)

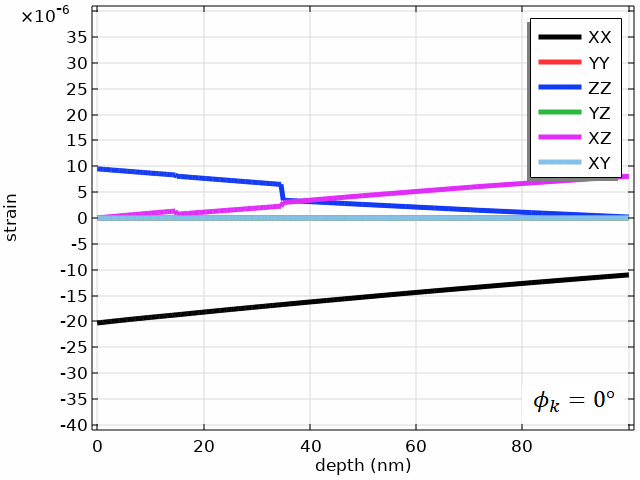

Supplement: Supplementary file 2 — Movies S1 to S4 [file sciadv.ado2504_movies_s1_to_s4.zip › ado2504_Movie_S1.gif]

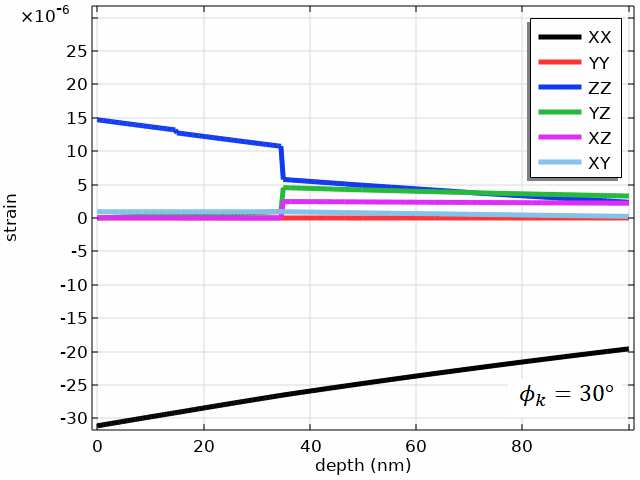

Supplement: Supplementary file 2 — Movies S1 to S4 [file sciadv.ado2504_movies_s1_to_s4.zip › ado2504_Movie_S2.gif]

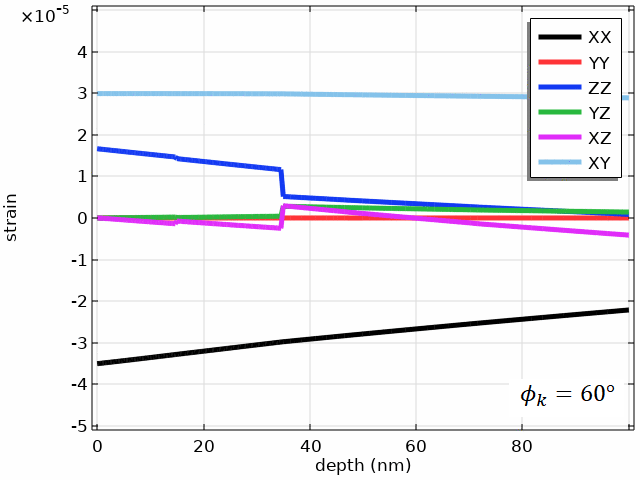

Supplement: Supplementary file 2 — Movies S1 to S4 [file sciadv.ado2504_movies_s1_to_s4.zip › ado2504_Movie_S3.gif]

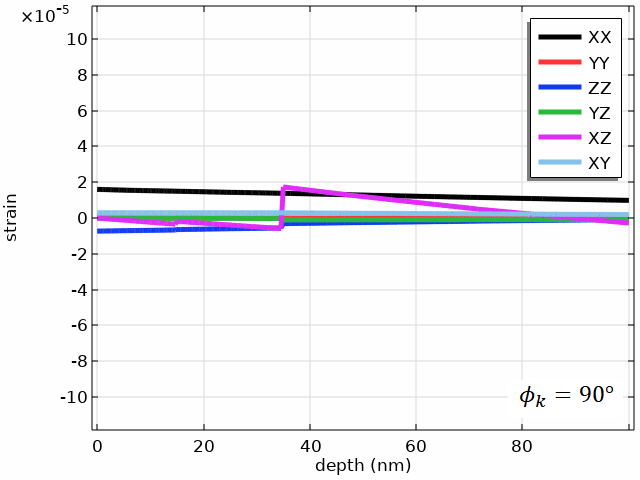

Supplement: Supplementary file 2 — Movies S1 to S4 [file sciadv.ado2504_movies_s1_to_s4.zip › ado2504_Movie_S4.gif]
